# Genome Sequence of a Segmented Filamentous Bacterium Strain That Confers a Rotavirus Resistance Phenotype in Mice

**DOI:** 10.1128/MRA.00073-20

**Published:** 2020-03-05

**Authors:** Zhenda Shi, Chunyu Zhao, Lisa M. Mattei, Kyle Bittinger, Baoming Jiang, Andrew T. Gewirtz

**Affiliations:** a Center for Inflammation, Immunity and Infection, Institute for Biomedical Sciences, Georgia State University, Atlanta, Georgia, USA; b Gastroenterology, Hepatology, and Nutrition, Children’s Hospital of Philadelphia, Philadelphia, Pennsylvania, USA; c Gastroenteritis and Respiratory Viruses Laboratory Branch, Division of Viral Diseases, Centers for Disease Control and Prevention, Atlanta, Georgia, USA; Queens College

## Abstract

Segmented filamentous bacteria (SFB) are well appreciated for eliciting Th17 cell immune responses. Here, we report the genome sequence of a murine isolate of SFB, which confers strong protection against rotavirus infection independent of acquired immunity.

## ANNOUNCEMENT

Segmented filamentous bacteria (SFB), are spore-forming bacteria that belong to the phylum of *Firmicutes*, in the order of *Clostridiales* (1, 2). SFB, which are frequently present in the mouse gut microbiota, are well known for impacting mucosal immune function, particularly driving the development of Th17 cells (3). Here, we report the whole-genome sequence of a newly isolated strain of SFB, namely, GSU-SFB, or SFB-G, which was discovered at Georgia State University (GSU).

The gut microbiota of a colony of Rag1-knockout (KO) mice housed at GSU, under IACUC approval, was observed to be uninfectable by rotavirus. Fecal transplant transferred rotavirus (RV) resistance to other mice. Analysis of the microbiota composition led to appreciation that RV resistance was associated with SFB (4). Hence, we isolated a strain of SFB present in these mice by the following approach. Fecal samples of RV-resistant mice were heated to 60°C for 10 minutes, followed by treatment of kanamycin (50 mg/ml) for 4 hours at 37°C. The resulting material was then transferred to germfree (GF) Rag1-KO mice by oral gavage. Recipient mice were RV resistant, and 95% of the total 16S rRNA marker gene sequences in their feces were annotated as SFB (4).

To assess the extent to which such SFB, namely, SFB-G, resemble previously described strains of SFB, we sequenced and analyzed it in parallel with a reference strain provided by Cerf-Bensussan and colleagues, referred to here as Pasteur-SFB (SFB-P). The SFB-P strain was also maintained by monoassociation of GF mice (5). DNA was extracted from feces and cecal contents of the SFB-mono-associated mice and sequenced on an Illumina HiSeq 2500 instrument, which generates paired 125-bp reads, using the Nextera XT DNA library preparation kit (Illumina, San Diego, CA). To increase genome coverage, sequences from 2 cecum samples, harvested from 2 littermate mice, and 1 fecal sample were combined for the assembly of SFB-G. The total numbers of raw reads generated for SFB-P and SFB-G are 19.6 million and 21.89 million, respectively. The raw sequencing reads were preprocessed using Sunbeam (version 1.0.2) (6), including trimming adapter sequences and removing the host genome. We then collected the so-called SFB reads by mapping reads to all 13 SFB genome sequences deposited in NCBI and extracted all the reads that aligned to any of the SFB genomes via BWA aligners (version 0.7.17). *De novo* assembly was carried out using SPAdes (version 3.11.1) (7) on the collected SFB reads, which resulted in 148 (*N*_50_, 17,773 bp) and 222 (*N*_50_, 7,628 bp) contigs for SFB-P and SFB-G, respectively. The size and G+C contents of the assembled genomes for SFB-P and SFB-G were 1,586,398 bp and 27.9% and 1,392,296 bp and 28.4%, respectively. The estimated completeness for our assembled genomes was 99.01% for SFB-P and 93.89% for SFB-G, as assessed using CheckM (version 1.0.7) (8). Sequences identified as SFB accounted for over 95% of all sequences, with other non-SFB microbial sequences displaying only very limited differences between SFB-G and SFB-P. SFB-G lacked 272 genes that were present in SFB-P and contained 133 genes not present in SFB-P and had an overall 12.2% smaller genome. The gene annotation was performed by Prokka (version 1.13), while pairwise proteome comparison was carried out using the PATRIC server (as of 2018 June) (9, 10). Genes that were identified as “bi (<->)” and “uni (->)” by PATRIC were designated to be shared and unique, respectively. Among the 133 genes contained by SFB-G, 28 of them were unique, i.e., not previously observed in any sequenced SFB isolates available in NCBI. A PATRIC analysis of genes whose presence differed between SFB-G and SFB-P revealed broad categories. Many of the unique genes in SFB-G were phage related, suggesting a potential contribution of phage-mediated anti-RV activities. Many genes absent in SFB-G were attributed to cell wall biosynthesis and modification, suggesting possible differences in the surface structures of this bacterium (4). The above analyses all used default software settings.

### Data availability.

The assembled SFB-P and SFB-G whole-genome sequences were deposited at GenBank under BioProject accession number PRJNA562885, BioSample accession number SAMN12657807, whole-genome sequence accession number WOUK00000000, and Sequence Read Archive accession number SRR10985095 (SFB-P) and BioProject accession number PRJNA562883, BioSample accession number SAMN12657733, whole-genome accession number WOUJ00000000, and Sequence Read Archive accession numbers SRR10985185, SRR10985186, and SRR10985187 (SFB-G).
